# Combined external beam radiotherapy with carbon ions and tumor targeting endoradiotherapy

**DOI:** 10.18632/oncotarget.25695

**Published:** 2018-07-06

**Authors:** Claudius Melzig, Azadeh Fahim Golestaneh, Walter Mier, Christian Schwager, Samayita Das, Julian Schlegel, Felix Lasitschka, Hans-Ulrich Kauczor, Jürgen Debus, Uwe Haberkorn, Amir Abdollahi

**Affiliations:** ^1^ German Cancer Consortium, Heidelberg, Germany; ^2^ Translational Radiation Oncology, National Center for Tumor Diseases, German Cancer Research Center, Heidelberg, Germany; ^3^ Division of Molecular and Translational Radiation Oncology, Heidelberg Institute of Radiation Oncology, National Center for Radiation Research in Oncology, Heidelberg, Germany; ^4^ Heidelberg Ion-Beam Therapy Center, Department of Radiation Oncology, Heidelberg University Hospital, Heidelberg, Germany; ^5^ Diagnostic and Interventional Radiology, Heidelberg University Hospital, Heidelberg, Germany; ^6^ Department of Nuclear Medicine, Heidelberg University Hospital, Heidelberg, Germany; ^7^ Clinical Cooperation Unit Nuclear Medicine, German Cancer Research Center, Heidelberg, Germany; ^8^ Department of Pathology, University Hospital Heidelberg, Heidelberg, Germany

**Keywords:** radioimmunotherapy (RIT), carbon ions, cetuximab, benzamide, immune response

## Abstract

External beam radiotherapy (EBRT) with carbon ions and endoradiotherapy using radiolabeled tumor targeting agents are emerging concepts in precision cancer therapy. We report on combination effects of these two promising strategies.

Tumor targeting ^131^I-labelled anti-EGFR-antibody (Cetuximab) was used in the prototypic EGFR-expressing A431 human squamous cell carcinoma xenograft model. A ^131^I-labelled melanin-binding benzamide derivative was utilized targeting B16F10 melanoma in an orthotopic syngeneic C57bl6 model. Fractionated EBRT was performed using carbon ions in direct comparison with conventional photon irradiation.

Tumor uptake of ^131^I-Cetuximab and ^131^I-Benzamide was enhanced by fractionated EBRT as determined by biodistribution studies. This effect was independent of radiation quality and significant for the small molecule ^131^I-Benzamide, i.e., >30% more uptake in irradiated vs. non-irradiated melanoma was found (p<0.05). Compared to each monotherapy, dual combination with ^131^I-Cetuximab and EBRT was most effective in inhibiting A431 tumor growth. A similar trend was seen for ^131^I-Benzamide and EBRT in B16F10 melanoma model. Addition of ^131^I-Benzamide endoradiotherapy to EBRT altered expression of genes related to DNA-repair, cell cycle and cell death. In contrast, immune-response related pathways such as type 1 interferon response genes (ISG15, MX1) were predominantly upregulated after combined ^131^I-Cetuximab and EBRT. The beneficial effects of combined 131I-Cetuximab and EBRT was further attributed to a reduced microvascular density (CD31) and decreased proliferation index (Ki-67).

Fractionated EBRT could be favorably combined with endoradiotherapy. ^131^I-Benzamide endoradiotherapy accelerated EBRT induced cytotoxic effects. Activation of immune-response by carbon ions markedly enhanced anti-EGFR based endoradiotherapy suggesting further evaluation of this novel and promising radioimmunotherapy concept.

## INTRODUCTION

External beam radiotherapy (EBRT) using heavy ions offers several advantages over conventional photon-irradiation e.g., high precision irradiation of tumor located deep in body or in proximity of organs at risk as well as enhanced relative biological effectiveness (RBE) [1, 2]. Recent reports on long-term follow-up data indicate that dose escalation is feasible with carbon ions resulting in excellent local control rates while sparing critical organs such as brain stem in the proximity of skull base tumors [3, 4]. Moreover, a growing body of data postulate successful eradication of otherwise radioresistant tumor subpopulations such as glioma stem cells and head and neck cancer stem cells in hypoxic niches [5, 6].

Endoradiotherapy (EndoRT) promises to selectively deliver radiation dose to the tumor tissue by tumor targeting agents conjugated with radionuclides. Dose-limiting toxicity is mainly defined by the characteristics of the tumor targeting agent, e.g., bone marrow suppression due to the long circulation times for radioimmunotherapy using antibodies or renal insufficiency due to fast renal clearance of small molecules/peptides [7]. This offers the opportunity to combine EBRT with EndoRT and thereby increase the total dose of tumor irradiation while diversifying radiation toxicity. Moreover, addition of systemic EndoRT treatment may assist to improve the local control of EBRT by efficiently eradicating locally invasive cells outside the high-dose radiotherapy field and improve overall outcome by targeting potential distant (micro)metastases.

In line with abovementioned rationales, promising results have been reported for the concept of combining photon-EBRT and EndoRT using different cancer entity specific targets, chemical moieties and radionuclides [8–14]. However, whether this concept also translates to beneficial outcomes in combination with high linear energy transfer (LET) carbon irradiation remains elusive. Moreover, the characteristic features of theragnostic agents, e.g., small molecule vs. immunoglobulin and consequently the EndoRT strategy that might be most beneficial for combined modality treatment with EBRT remain to be elucidated. Therefore, we investigated the effects of combined modality treatments using endoradiotherapy in two tumor-targeting models with carbon ion or conventional photon EBRT, respectively.

Head and neck squamous cell carcinoma (HNSCC) is an entity for which radiotherapy is a central part of oncologic management [15, 16]. Elevated tumor EGFR expression level is linked to tumor aggressiveness and radioresistance constituting a major challenge for successful therapy of locally advanced HNSCC [17]. Therefore, dual modality therapy consisting of anti-EGFR and radiotherapy was rationally designed utilizing Cetuximab, a chimeric IgG1 antibody, and this was the first combination of targeted therapy with EBRT to receive FDA-approval for the treatment of locally advanced HNSCC [18]. Based on this success different approaches for development of anti-EGFR based theragnostics was studied using different antibodies, chelators and radionuclide combinations for radioimmunotherapy of EGFR expressing tumor entities [19–26]. Further, preclinical rationales for combined anti-EGFR EndoRT with EBRT were developed [13, 27–29].

Despite recent advances with BRAF- and immune checkpoint-inhibitors in metastastic melanoma about half of the patients do not respond initially and even more ultimately progress after a transient response [30–33]. Radiotherapy plays a critical role in management of melanoma patients at an oligometastatic stage, i.e., to control frequently appearing brain metastases as well as extracranial (SABR/SBRT) treatment of symptomatic metastases in lung and lymph nodes [34, 35]. Since the first description of benzamide derivatives as promising melanin targeting molecules [36] several compounds have been developed to improve pharmacological properties of benzamide based theragnostics in metastatic melanoma. Recently, we reported on dosimetry and first therapeutic experiences in 9 patients with metastatic melanoma using a ^131^I-labeled benzamide [37]. A preclinical study by Joyal et al. demonstrated a promising therapeutic efficacy of a newly developed benzamide labeled with ^131^I, MIP-1145 [38]. To our knowledge, the present manuscript is the first to investigate the combination of melanin-targeting EndoRT (^131^I-Benzamide) and EBRT in the melanin expressing syngeneic B16F10 melanoma model. Moreover, carbon-EBRT combined with anti-EGFR based EndoRT was studied using ^131^I-Cetuximab in a prototypic EGFR amplified A431 human squamous cell carcinoma xenograft model.

## RESULTS

To examine the impact of EBRT with photon and carbon ions on tumor uptake kinetics of small molecule (benzamide) and antibody (Cetuximab) based EndoRT, we first investigated the biodistribution of both compounds as a function of EBRT.

### Tumor uptake of ^131^I-Cetuximab and ^131^I-MIP-1145 in irradiated and non-irradiated tumors

**Figure 1:**
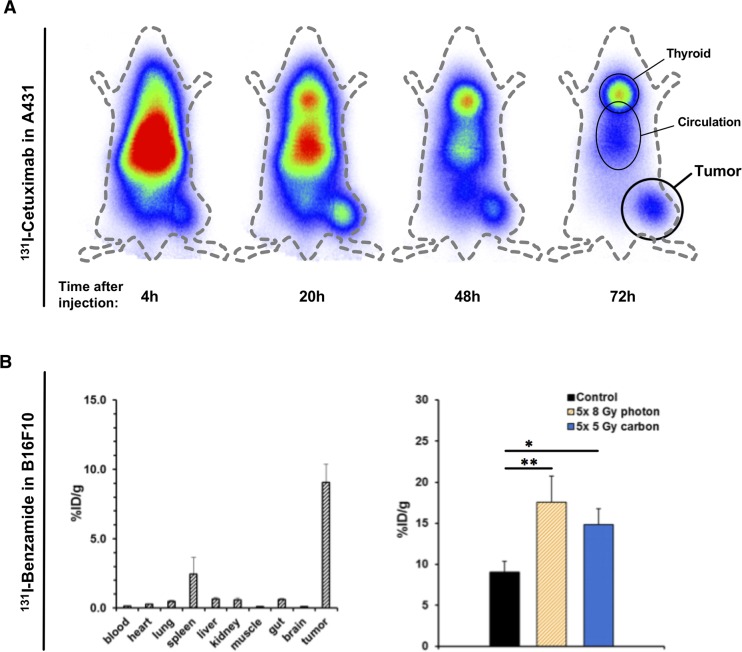
Effect of irradiation on tumor uptake of Iodine-labelled Cetuximab and Benzamide **(A)** An A431-bearing nude mouse was injected with ^131^I-labeled intravenously and radioactivity distribution assessed over time using a gamma camera. **(B)** *In vivo* biodistribution of ^131^I-Benzamide was assessed 24h after intravenous injection in untreated B16F10-bearing mice (left). To analyze the effect of prior irradiation on tracer uptake animals underwent EBRT first and tracers were injected on the third day after the last fraction (right). EBRT-doses were 5x 8 Gy photon or 5 Gy carbon daily. Again, organ distribution was measured 24h after tracer injection. Data points indicate mean ± SEM ^*^: p-value < 0.05, ^**^: p-value < 0.01.

Tumor targeting and biodistribution of ^131^I-Cetuximab in A431 and ^131^I-Benzamide in B16F10 was assessed using Gamma-camera imaging and *in vivo* biodistribution experiments (Figure 1 and Supplementary Figure 1). A gamma camera time series of ^131^I-Cetuximab in an A431 bearing mouse revealed peak accumulation of the labelled antibody in tumor 1 day after injection (Figure 1A).

An *in vivo* biodistribution assay was conducted in the syngeneic B16F10-model with ^131^I-Benzamide (Figure 1B, left). The observed tumor uptake 24h p.i. was 9.0 ± 4.2 %ID/g, tumor-to-muscle ratio (TMR) was 107.6 (n = 11). Uptake by other organs was relatively low (spleen 2.4 ± 4.0 %ID/g, liver 0.6 ± 0.3 %ID/g, kidney 0.5 ± 0.5 %ID/g, lung 0.5 ± 0.2 %ID/g) and comparable to previously published data in human [37]. The same biodistribution assay was performed with ^131^I-labeled Cetuximab in mice with subcutaneous A431-tumors at 24h p.i. (Supplementary Figure 1A). ^131^I-Cetuximab uptake in the tumor was 3.6 ± 1.4 %ID/g with a TMR of 5.2. Uptake was also high in lung (6.5 ± 2.3 %ID/g) and liver (4.1 ± 1.9 %ID/g).

To explore the effect of radiotherapy on tumor theragnostic uptake, after EBRT animals were injected with ^131^I-Cetuximab or ^131^I-Benzamide, respectively. In B16F10-bearing mice organ distribution on day 3 after irradiation with 5 consecutive daily fractions of 8 Gy photon or 5 Gy carbon-EBRT, respectively, revealed a significantly enhanced tumor-enrichment (Figure 1B, right): Tumor-uptake reached 17.5 ± 4.5 %ID/g, TMR 195.1 (p-value 0.01; n = 3) after photon-EBRT and 14.8 ± 2.0 %ID/g, TMR 161.5 (p-value 0.029; n = 4) after carbon-EBRT. EBRT with 5 daily fractions of 3 Gy photon or 1 Gy carbon, respectively, also increased the uptake of ^131^I-Cetuximab in A431 tumors to 4.4 ± 1.9 %ID/g after photon and 4.4 ± 4.2 %ID/g after carbon irradiation although not to the level of statistical significance (Supplementary Figure 1B).

### Tumor growth delay *in vivo* under combined EBRT and ^131^I-Cetuximab endoradiotherapy

The efficacy of a sequential combined therapy with endoradiotherapy and photon-EBRT (PERT) or carbon ion-EBRT (CERT) was assessed by following the same treatment schedule as for biodistribution experiments.

**Figure 2:**
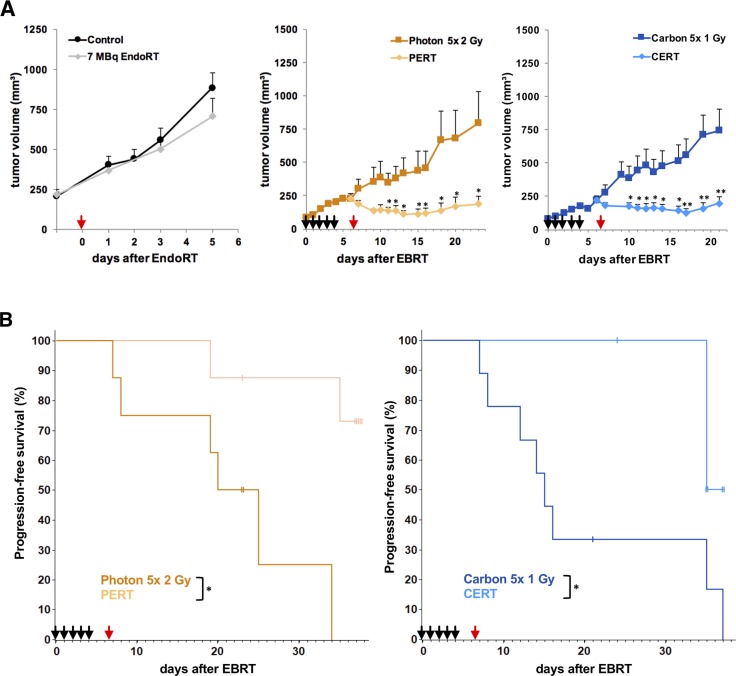
Combined treatment with EBRT and 131I-Cetuximab improves A431 tumor growth inhibition and progression-free survival Animals were treated with 5 daily fractions of EBRT (black arrows; 2 Gy photon or 1 Gy carbon per fraction), a single fraction of 7 MBq ^131^I-Cetuximab (red arrow) or a combination of the two. Untreated animals served as controls. Data points indicate mean tumor-volumes ± SEM. **(A)** Reaching a tumor volume of 500 mm^3^ was considered tumor progression and defined as an event for Kaplan-Meier-analysis. **(B)** ^*^: p-value < 0.05, ^**^: p-value < 0.01.

By the time A431-xenograft tumors had reached a size of 86 ± 6 mm^3^ the tumors were irradiated with five daily fractions of 1 Gy physical dose carbon ion-irradiation or 2 Gy photon-irradiation, respectively (Figure 2A). By the time of administration of 7 MBq ^131^I-Cetuximab endoradiotherapy tumor sizes of A431 were 211 ± 21 mm^3^, 229 ± 33 mm^3^, 221 ± 33 mm^3^ in the control, photon-EBRT and carbon-EBRT group. Untreated control animals reached a high tumor burden within 5 days and had to be sacrificed. Compared to size-matched controls, endoradiotherapy alone led to a non-significant reduction in tumor volume of 20% on day 5 after the administration of ^131^I-Cetuximab (p > 0.2).

Photon- or carbon-EBRT alone led to a highly significant tumor growth inhibition as of day 1 after the beginning of treatment compared to size-matched controls (p < 0.01). Mean tumor sizes were reduced by 60% and 71% on day 5 after the start of photon- or carbon-EBRT, respectively.

The effect of combined modality treatment on tumor growth delay compared to EBRT alone was statistically significant as of day 4 after endoradiotherapy, i.e. day 11 after EBRT in the PERT group and as of day 3 after endoradiotherapy, i.e. day 10 after EBRT in the CERT group (p < 0.05). On day 5 after the administration of EndoRT, i.e. 12 days after EBRT, mean tumor sizes were reduced by 64% and 67% in the PERT and CERT group, respectively, compared to each EBRT alone.

Kaplan-Meier estimate was utilized to compare time to progression, i.e., 5-fold increase of tumor size to a volume of 500 mm^3^ was considered as event (Figure 2B). Median time to progression was 20 days in the photon-EBRT and 15 days in the carbon-EBRT group. By the end of the study, on day 38 after treatment start, 75% of the animals in the PERT group and 65% of the animals in the CERT group were event-free (p < 0.05). There was no significant difference in mean tumor size or time to progression between photon- and carbon-EBRT groups. Hence, in terms of tumor growth inhibition, 10 Gy photon and 5 Gy carbon in five consecutive daily fractions were iso-effective.

### Tumor growth delay *in vivo* under combined EBRT and ^131^I-Benzamide in B16F10 tumors

**Figure 3:**
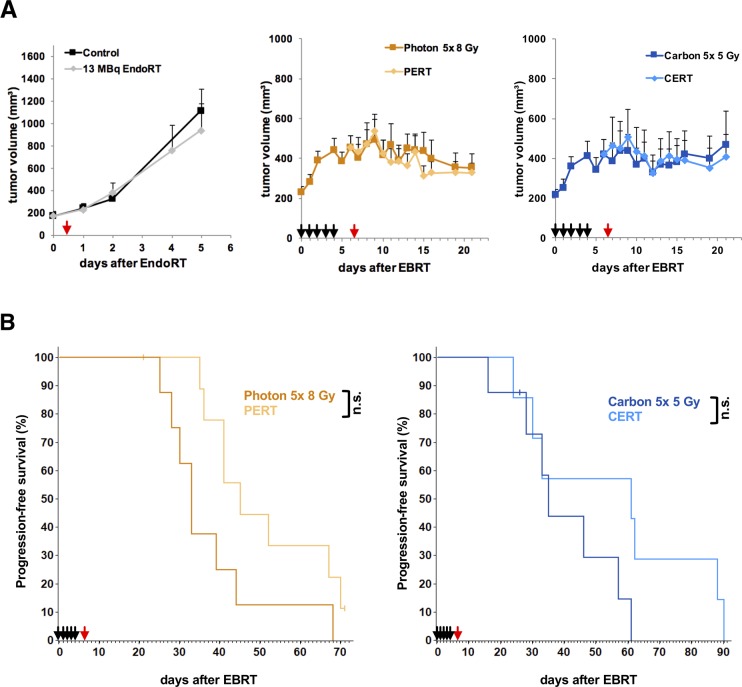
Tumor growth delay and time to progression of B16F10-tumors under treatment with EBRT and 131I-Benzamide Animals were treated with 5 daily fractions of EBRT (black arrows; 8 Gy photon or 5 Gy carbon per fraction), a single fraction of 13 MBq ^131^I-Benzamide (red arrow) or a combination of the two. Untreated animals served as controls. Data points indicate mean tumor-volumes ± SEM **(A)** Reaching a tumor volume of 1000 mm^3^ was considered tumor progression and defined as an event for Kaplan-Meier-analysis. **(B)** ^*^: p-value < 0.05, ^**^: p-value < 0.01.

B16F10 tumors measured 225 ± 19 mm^3^ by the beginning of EBRT with 5 daily fractions of either 8 Gy photon- or 5 Gy carbon-irradiation each. By the time 13 MBq ^131^I-Benzamide EndoRT was administered tumors measured 455 ± 57 mm^3^ in the photon- and 419 ± 77 mm^3^ in the carbon-EBRT group (Figure 3A). Tumors in the EndoRT alone group were treated at a mean tumor volume of 174 ± 21 mm^3^. Tumors in the control and EndoRT group showed rapid growth and animals had to be sacrificed 5 days after EndoRT treatment.

EBRT led to significant tumor growth inhibition compared to untreated controls by 61% in the photon-EBRT group (440 ± 60 mm^3^ vs. 1117 ± 190 mm^3^, p < 0.01) and 63% in the carbon-EBRT group (414 ± 70 mm^3^ vs. 1117 ± 190 mm^3^, p < 0.01) on day 4 after EBRT. There was no significant difference in tumor growth inhibition between the 40 Gy total dose photon- and 25 Gy total dose carbon-EBRT (p > 0.5). EndoRT alone had no significant impact on tumor growth (1117 ± 190 mm^3^ vs. 938 ± 237 mm^3^, p > 0.5).

We observed no significant tumor growth inhibition in PERT and CERT groups compared to the respective EBRT alone (p > 0.1).

Kaplan-Meier-analysis of time to progression, i.e., a 5-fold increase in tumor size to a volume of 1000 mm^3^ was considered an event, showed a trend to be improved under combined treatment but did not reach statistical significance (Figure 3B): median time to progression was 33 days in photon-EBRT vs. 45 days in PERT group (p = 0.054) and 35 days in carbon-EBRT vs. 61 days in CERT group (p = 0.18).

The B16F10 tumor cells used in this model are stably transduced with lentiviral vector and selected for high luciferase expression allowing bioluminescence imaging (Figure 7A). Bioluminescence imaging 5 weeks after the beginning of treatment revealed significantly lowered luciferase activity in CERT group compared to carbon-EBRT alone by 88% (p < 0.05). A trend towards lowered luciferase activity of 70% was also observed in PERT compared to photon-EBRT but did not reach statistical significance (p = 0.2).

### Effects of combined treatment on tumor microvasculature and proliferation

Tissue samples for histology and transcriptome analysis were all collected at the same time point 5 days after EndoRT or 8 days after the last fraction of EBRT.

**Figure 4:**
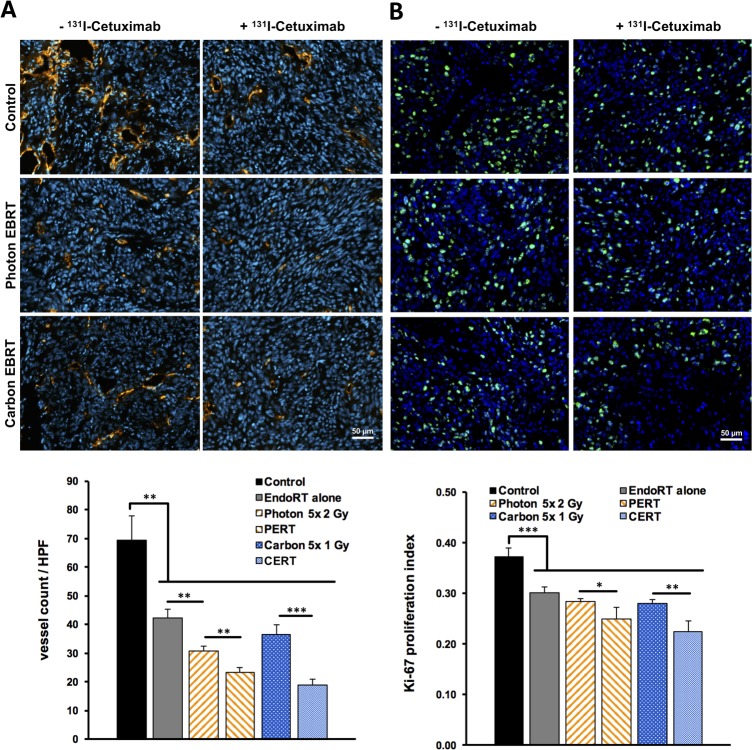
Reduced tumor proliferation and microvascular density after combined EBRT and 131I-Cetuximab endoradiotherapy Immunohistochemistry of tumor sections one week after EBRT or 5 days after endoradiotherapy. **(A)** Microvessel density was analyzed by immunofluorescent staining for endothelial CD31 (red). DAPI was applied as nuclear co-staining (blue). The number of vessels per high-power field on each section was counted automatically. **(B)** Proliferation index of A431 tumors under therapy was assessed by staining tumor sections for Ki-67 (green) and using DAPI as nuclear co-staining (blue). Ki-67 positive cells per high-power field were counted automatically and proliferation indices were calculated. Data points indicate mean ± SEM. ^*^: p-value < 0.05, ^**^: p-value < 0.01.

Treatment-induced changes to the tumor microvasculature were investigated by staining snap-frozen sections of A431 tumors for CD31 (Figure 4A, Supplementary Figure 5). We found a significant reduction in microvessel density (MVD) in the combined treatment groups compared to each monotherapy: MVD was reduced by 24% in PERT compared to photon-EBRT alone (p < 0.01) and by 48% in CERT compared to carbon-EBRT alone (p < 0.001). There was a trend towards ∼ 20% decreased MVD between CERT vs. PERT, which did not reach statistical significance (p = 0.07). Endoradiotherapy alone also significantly reduced MVD compared to the control group by 39% (p < 0.01).

We evaluated the effects of treatment regimen on tumor cell proliferation by staining tumor sections for the proliferation marker Ki-67 (Figure 4B). EndoRT as well as each EBRT alone significantly reduced tumor cell proliferation compared to untreated controls (p < 0.001). Compared to each EBRT alone ^131^I-Cetuximab based PERT or CERT significantly lowered the proliferation index by 12% (p < 0.05) and 20% (p < 0.01), respectively.

### Carbon ion irradiation augmented immune response to ^131^I-Cetuximab

**Figure 5:**
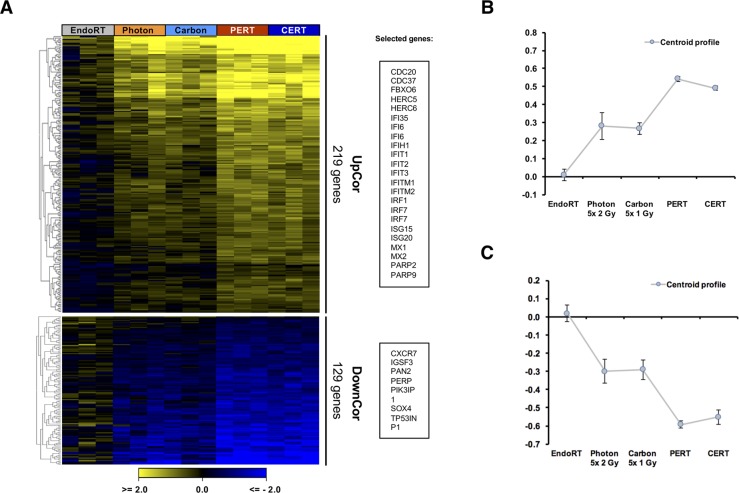
Therapy dependent gradual regulation of gene expression in A431 tumor One week after EBRT or 5 days after endoradiotherapy tumor tissue was collected and processed for genome-wide expression analysis. Treatment intensification correlated with a gradual up- or downregulation of genes (UpCor and DownCor) resulting into a 3-step profile from endoradiotherapy only over EBRT only to combined treatment. **(A)** Heatmap of genes significantly correlating (yellow) or anti-correlating (blue) with Up-/Down-Cor profile. A correlation coefficient of r ≥ 0.8 (i.e. p < 0.001) was chosen as cut-off. Genes were clustered according to Euclidian distance. The corresponding centroid profiles **(B** and **C)** mirror the selected template profile.

To investigate tumor therapy responses on molecular level, genome-wide transcriptional analysis was performed. Hierarchical clustering using euclidian distance and average linkage analysis of 500 most significantly regulated genes (ANOVA, p<0.01) in A431 model across all therapy modalities revealed a gradual expression pattern from EndoRT to EBRT and C/PERT as a predominant profile (Supplementary Figure 2). Next, we searched for genes demonstrating this gradual profile using a supervised approach, i.e. expression pattern of all genes was correlated against the pre-defined template with a gradual up-/down regulation profile (Pavlidis Template Matching, PTM). Of the 26307 transcripts analyzed in A431, 219 were gradually upregulated (UpCor) and 129 downregulated (DownCor) with a correlation coefficient of r ≥ 0.8 (p < 0.001 Figure 5). Less stringent cut-off criteria (r ≥ 0.7, p < 0.01) resulted into 658 UpCor and 444 DownCor transcripts (Supplementary Figure 3).

**Figure 6:**
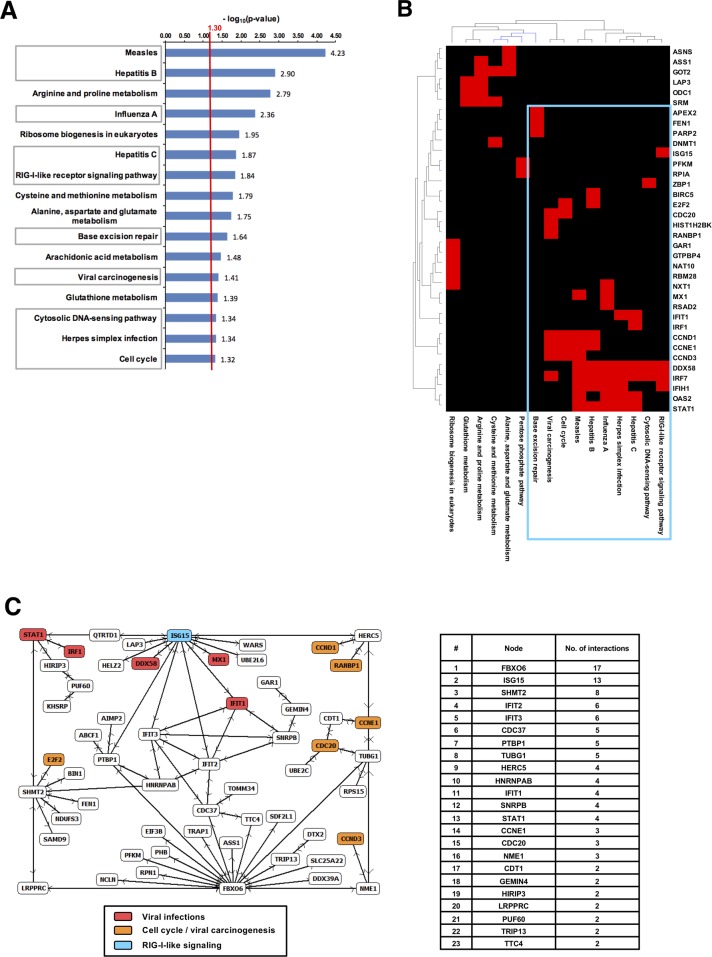
Combined Cetuximab endoradiotherapy and EBRT induced a potent immune response **(A)** Pathway enrichment analysis among UpCor genes identified immune response associated categories to be significantly affected by combined anti-EGFR and EBRT. A population map highlights clusters of upregulated genes that significantly enriched KEGG-pathways have in common. **(B)** A gene regulatory network was identified considering only direct known interaction among the selected UpCor genes. Color-coding of gene-names indicates participation in one of the significantly enriched KEGG-pathways in and the degree of connection of each single pathway component **(C)**.

Within the set of gradually expressed genes to the level of p < 0.001 we searched for significantly affected biochemical pathways by performing a gene set enrichment analysis using the KEGG-database. Among the significantly enriched KEGG-pathways in the UpCor list, immune response related processes such as infectious diseases, DNA-damage as well as Cell-cycle control pathways were found (p < 0.05, Figure 6). Clustering of genes involved in enriched pathways indicated relevance of a few genes that were common across e.g. immune response related pathways (Figure 6B). For example, STAT1, PARP2, Cyclin D1 (CCND1), D3 (CCND3) and E1 (CCNE1) and CDC20 were found among the UpCore genes. In contrast, DownCor genes were mainly enrichment for metabolic pathways as well as the Hippo signaling pathway (Supplementary Figure 4). A less restrictive gene selection by choosing r ≥ 0.7 (p < 0.01) as cut-off identified more metabolic pathways, particularly in amino-acid and carbohydrate metabolism to be enriched (Supplementary Figure 3A).

We then searched for protein-protein interactions and gene-regulatory networks among correlating genes utilizing NCBI human protein interaction database. Within the UpCor gene set, six gene-regulatory networks consisting of two or more nodes were identified. The largest network consisted of 57 nodes without interpolation, i.e., 26% of all UpCor transcripts were directly interconnected by known interactions (p < 0.005). The highest-ranking hub-node was F-box only protein 6 (FBXO6), a member of ubiquitin protein ligase complex with 17 direct neighbors within this network. The second largest hub node was Interferon-stimulated gene 15 (ISG15), a central player of type I interferon response [39, 40] and member of the RIG-I-like receptor signaling pathway which was also found to be enriched in the gene set enrichment analysis (Figure 6A and 6B). Other members of the network also reappeared in the gene set enrichment analysis as members of pathways associated with viral infections (DDX58, IFIT1, IRF1, MX1, STAT1) or viral carcinogenesis and cell cycle (CCND1, CCND3, CCNE1, CDC20, E2F2, RANBP1).

Within the DownCor gene set we identified 15 networks of which the largest consisted of 63 nodes or 14% of all DownCor genes (p < 0.003 Supplementary Figure 3B). The key hub-node, PAN2 had 16 direct interactions. Two other members of the network, PPP1CB and PPP2CB, are part of the Hippo signaling pathway that was enriched in the gene set enrichment analysis. 7 other proteins, 11% of the network, belong to the KEGG-pathway ‘ribosome’.

### Enrichment of cell cycle and p53 related genes after combined modality treatment in B16F10 melanoma model

**Figure 7:**
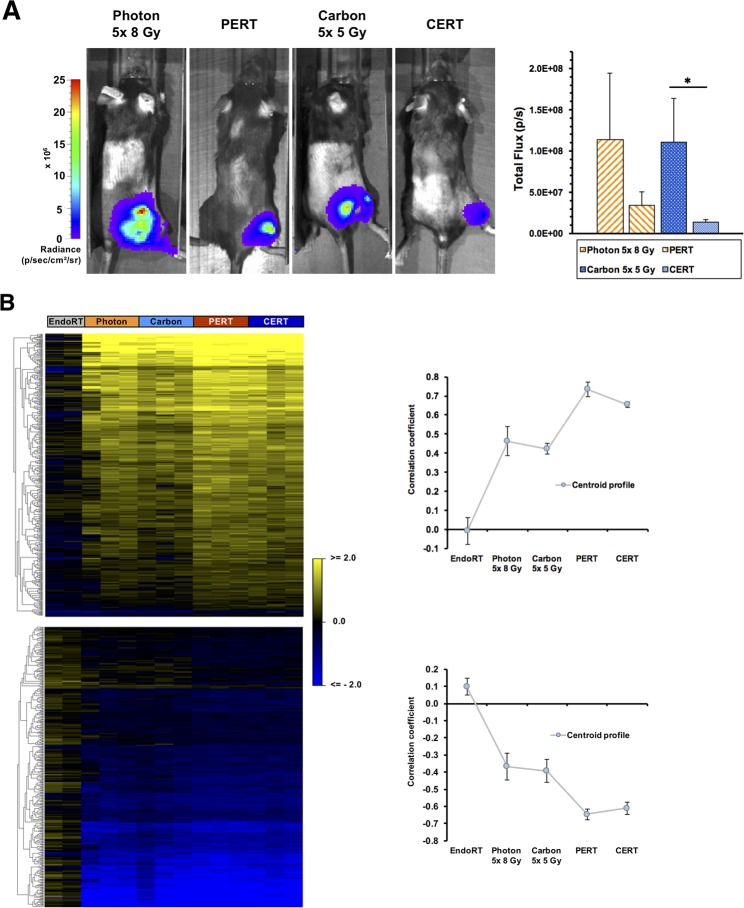
Treatment effects of combined EBRT and 131I-Benzamide endoradiotherapy on Bioluminescence and gene expression in B16F10 luc2+-bearing mice **(A)** Bioluminescence imaging of B16F10-bearing mice 5 weeks after the initiation of EBRT. Data points indicate mean ± SEM ^*^: p-value < 0.05. Transcriptome profile of B16 tumors under therapy. **(B)** Heatmap (left) of genes correlating or anti-correlating with a gradual therapy intensification and a correlation coefficient of at least r ≥ 0.7 (p < 0.01). Genes were clustered according to their Euclidian distance. The corresponding cluster centroid profiles (right).

**Figure 8:**
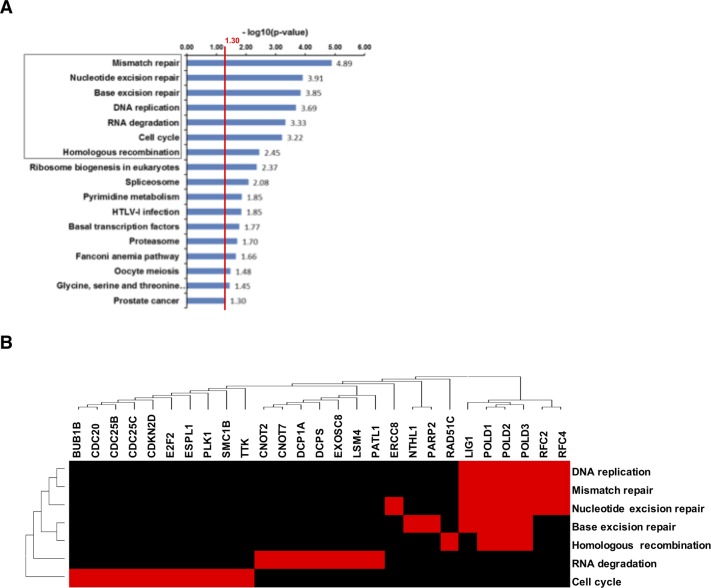
Down-regulation of genes related to DNA-damage response in B16F10 tumors after dual therapy **(A)** KEGG-pathways were searched for enrichment of DownCor genes at the level of r ≥ 0.7 (p < 0.01). All significantly enriched KEGG-pathways are presented here. The red line indicates the cut-off for statistical significance for pathway enrichment to the level of p < 0.05. A population map highlights clusters of upregulated genes that the enriched KEGG-pathways have in common **(B)**.

**Figure 9:**
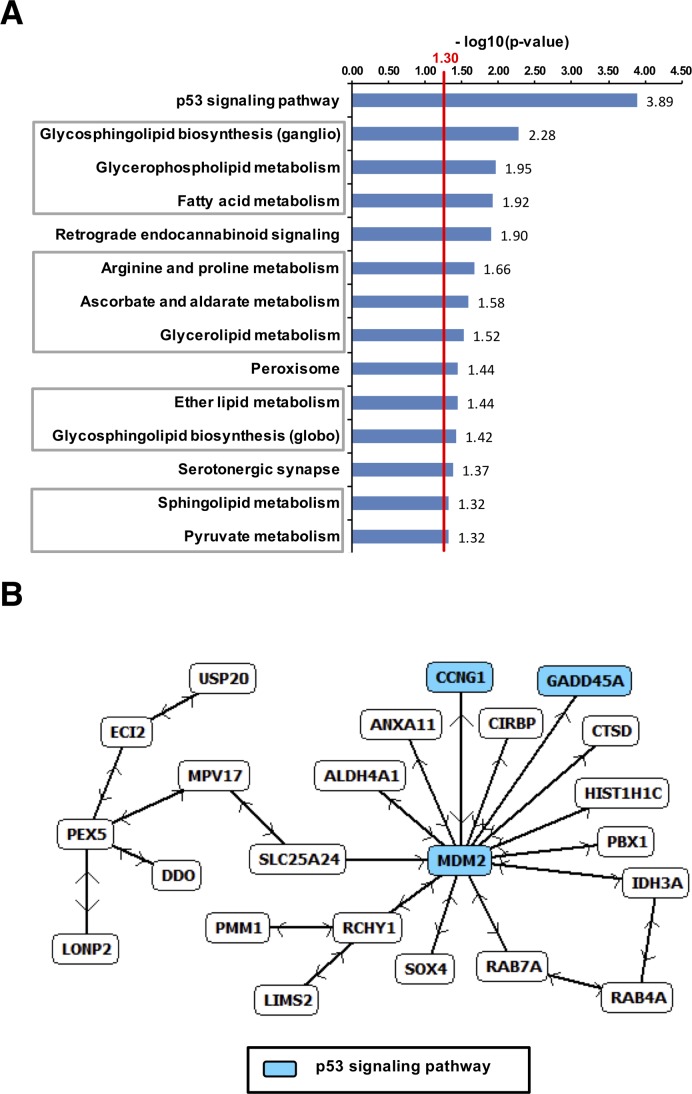
Upregulated genes in B16F10-tumors after dual treatment **(A)** Pathway enrichment by UpCor genes (r ≥ 0.7, p < 0.01). The red line indicates the cut-off for statistical significance for pathway enrichment to the level of p < 0.05. The largest direct interaction network among the positively correlating genes was identified and again contained p53 associated genes **(B)**.

To dissect the tumor response mechanisms genome-wide expression profiling was also applied to B16F10 tumor specimen collected from each therapy arm (Figures 7-9). Applying the same approach described for A431-tumors, 448 UpCor and 406 DownCor genes were found in the melanoma model to be gradually regulated (r ≥ 0.7, p < 0.01, Figure 7B). Pathway enrichment analysis in the UpCor gene set identified p53 signaling as the pathway with the strongest representation among the positively correlating genes (Figure 9A). Genes in the UpCor set involved in p53 signaling were: Ccnd1, Ccnd2, Ccng1, Ei24, Gadd45a, Mdm2, Rchy1, Zmat3. Other significantly enriched pathways can be summarized under intermediary lipid metabolism. The largest direct interactions network within the UpCor gene set consisted of 23 nodes (Figure 9B). Mouse double minute 2 homolog (MDM2), a member of the p53 signaling pathway is the largest hub node of this network with 13 neighbors. Two other members of this network, CCNG1 and GADD45A are also part of the p53 signaling pathway.

Within the DownCor gene set pathways mainly associated with DNA damage repair were significantly enriched (Figure 8) with the most significantly enriched ones being mismatch repair (MMR), nucleotide excision repair (NER) and base excision repair (BER), homologous recombination (HR), DNA-replication and RNA-degradation.

## DISCUSSION

We report on enhanced relative biological efficacy (RBE) of radiotherapy with carbon ions vs. conventional radiotherapy with photons in two in-vivo models. In A431 xenograft model, similar tumor growth delay efficacy was achieved after 5×1Gy carbon ion vs. 5×2Gy photon irradiation (RBE ∼ 2), whereas 5×5Gy carbon ion was comparable with 5×8Gy photon irradiation in the syngeneic B16F10 melanoma model (RBE ∼ 1.6). Moreover, to our knowledge, first data on successful combination of carbon ion EBRT and EndoRT (CERT) utilizing ^131^I-labelled monoclonal anti-EGFR Cetuximab in A431 and small molecule approach with ^131^I-Benzamide in B16F10 melanoma model is reported.

Biodistribution experiments demonstrated successful targeting of the tumor in both models. Compared to data found in the literature our biodistribution experiments with ^131^I-Cetuximab showed a lower absolute tumor-uptake in non-irradiated A431 tumors of 3.6 %ID/g. Ping Li et al. for example saw a tumor-uptake of 18.5 %ID/g of ^64^Cu-DOTA-Cetuximab in A431 at 24 hours p.i. [25]. On the other hand, the TMR of 5.2 that we observed at 24h together with the biodistribution profile suggesting relatively elevated uptake in lung and liver was in line with the published literature [23, 25]. Cetuximab binds to EGFR on A431 and is known to be internalized [23, 41]. Radioiodinated Cetuximab in our study contained a non-residualizing iodotyrosine (^131^I) moiety which has been shown to rapidly wash out from the cells post internalization [42]. Together, these could provide plausible explanations for the observed thyroid uptake and the relative lower A431 uptake of ^131^I-Cetuximab in our study compared to previously reported approaches utilizing residualizing chelator-radionuclide complexes. Therefore, more stable chelator based radiolabeling should be favored for clinical application of Cetuximab based endoradiotherapy [19–26]. In the B16F10-model we utilized a small molecule, MIP-1145, belonging to the chemical class of benzamide-derivatives which binds specifically to melanin [36]. ^131^I-MIP-1145 well accumulated in B16F10 melanoma, with 9 %ID/g and a TMR of 107.6. This is in accordance with previous data in SKMEL-3 melanoma xenograft model reported by Joyal et al. demonstrating a tumor uptake of 5.91 ± 3.94 %ID/g and a TMR of about 197 for ^131^I-MIP1145 at 24 hours after injection [38].

Another important finding is that EBRT, independent of radiation quality, led to an enhancement in tumor-uptake, especially in B16F10 where an up to 81% increase of TMR was observed. The radiation induced enhanced uptake of the small molecule MIP-1145 is in line with previous reports attributing an improved delivery of nanomedicine such as liposomal doxorubicin to the irradiated tumor [43]. To our knowledge, there are no data on the influence of irradiation on tumor uptake of Cetuximab. Controversies exist on the impact of radiation dose, fractionation and scheduling/sequencing on reduced or enhanced uptake of antibodies and macromolecules [44, 45]. While, in long-term, antiangiogenic and reduced tumor perfusion effects of EBRT may limit the uptake of macromolecules, there might be a therapeutic window during or after fractionated irradiation where tumor perfusion is improved by irradiation e.g. via pruning of therapy sensitive immature vessels and reduced intratumoral interstitial fluid pressure [46, 47]. Indeed previous tumor biodistribution experiments with copolymers of different sizes showed a wide variety of tumor uptake-enhancement ranging from 11% to 105% 24 hours after irradiation [45]. Our data support this observation and indicate a therapeutic window for enhanced delivery of endoradiotherapy after fractionated irradiation.

Tumor growth delay was significantly enhanced by addition of ^131^I-Cetuximab to fractionated EBRT. By the end of the study ∼ 70% of tumors in the CERT/PERT groups were still controlled, whereas all tumors in the single treatment groups had already shown a doubling of volume. The reduction of tumor proliferation and microvessel density mirrored tumor therapy responses. These data are in line with previous reports for combined photon-EBRT and different Cetuximab based endoradiotherapies. For example, tumor control dose 50% (TCD_50_) with a dose-modifying factor of 3.73 was reported for administration of 2.5 MBq ^90^Y-labelled Cetuximab endoradiotherapy 2 to 4 days after a single dose of 8.3 Gy photon-EBRT in FaDu xenograft model [13]. Our data further expand these observations demonstrating a clear benefit for dual combination consisting of EGFR-targeting endoradiotherapy and carbon ion EBRT.

In syngeneic B16F10 model utilized here, monotherapy with 13 MBq ^131^I-MIP1145, despite marked tumor uptake, did not result in significant tumor growth inhibition. This finding is in contrast to previous report in SKMEL-3 melanoma xenograft model where treatment with a single dose of 25 MBq ^131^I-MIP1145 led to a significant tumor growth inhibition of 79% after 35 days compared to sham-treated controls [38]. In our syngeneic setting administration of 25 MBq ^131^I-MIP1145 was not well tolerated in C57bl6 mouse (data not shown), hence, we utilized a reduced dose. In addition, treatment was started at a relatively large tumor volume in this fast growing and aggressive B16F10 melanoma model, which may further explain the lack of efficacy of endoradiotherapy alone in our study. EBRT was effective in delaying tumor growth and assessment of tumor cell viability by bioluminscence imaging demonstrated a significant decrease in luciferase activity after CERT compared to carbon-EBRT alone. Although CERT translated into a trend towards an improved time to progression, statistical significance was not reached for both dual therapies vs. EBRT alone in B16F10 model. One plausible explanation for the observed discrepancy between bioluminescence vs. caliper measurement might be that the latter resembles a sum of heterogeneous processes affecting tumor volume, i.e. does not discriminate between inflammatory response, evolution of necrotic regions etc. compared to a thorough tumor cell proliferation derived volume growth. In contrast, the luciferase activity could be considered as a surrogate for the abundance of live and metabolic active tumor cells. The longer follow-up due to significant tumor growth delay after EBRT allowed observation of sporadically evolving B16F10 lymphnode (LN) metastases. However, no differences were found in frequency of LN metastases after CERT/PERT vs. EBRT alone suggesting no additional systemic benefit of administrating benzamide endoradiotherapy (data not shown). Transcriptome analysis revealed a gradual regulation of genes from ^131^I-MIP1145 to EBRT to P/CERT (UpCor and DownCor). Interestingly, DNA-damage response (DDR) and cell-cycle control genes were enriched for UpCor genes indicating enhanced radiation induced cytotoxicity after combined endo- and external radiotherapy. Among the UpCor genes p53 pathway was most significantly enriched (p≤0.0001). Moreover, MDM2 was the major hub of the identified UpCor gene-regulatory network linking known players of p53 signaling such as GADD45A to dual combination effect after ^131^I-MIP1145 and EBRT. GADD45 is among the few consistently reported overexpressed genes after irradiation [48]. GADD45 and MDM2 were also among the first genes to be identified as differentially regulated upon irradiation [49]. In addition to the known impact of MDM2 in limiting cell cycle arrest and initiation of programmed cell death, p53-independent role of this protein in orchestrating inflammatory response signaling might be of relevance for interpreting its relevance within the UpCor gene-regulatory network [50, 51]. Of note, A431 cell line carries a mutated TP53 gene [52, 53] resulting in a loss of function of p53. This might in part explain the lack of enrichment for p53 pathway in A431 in contrast to B16F10. Intriguingly, DownCor genes were significantly enriched for genes attributed to cellular DNA repair machinery i.e., MMR, NER, BER and HR, which are considered backup or alternative pathways to non-homologous end joining (NHEJ), the central DDR pathway for repair of radiation induced DNA double strand breakages (DSB). Therefore, downregulation of these DDR genes e.g., RAD51C, LIG1 and POLD1-3 may produce synergies towards formation of persistent unrepairable complex DSB in tumor cells treated with dual combination leading to cell growth arrest and lethality. Together, addition of endoradiotherapy with ^131^I-MIP1145 seems to enhance EBRT via further activation of genuine hallmarks of cellular radiotoxicity i.e., in direction of more of the same but via different routes of administration. In contrast, the combination of EBRT with EGFR targeting endoradiotherapy was found to activate syngergistic principles for tumor eradication.

Targeting EGFR has emerged as a promising strategy in EGFR amplified or addicted tumors. However, the mechanism of action of antibody-based strategies seems to differ substantially from pure inhibition of EGF receptor tyrosine kinase (RTK) activity which is chiefly targeted by small molecule RTK inhibitiors (RTKi). This may in part explain differential responses of tumors, e.g. with respect to local tumor control, after combined radiotherapy and RTKi vs. anti-EGFR [54]. Moreover, differences in clinical outcome are observed between the two EGFR targeting antibodies, Panitumumab and Cetuximab indicating that beyond EGFR targeting also the property of the immunoglobulin Fc moiety to induce antibody dependent cellular cytotoxicity (ADCC) matters [55]. Indeed, as an IgG1 antibody, Cetuximab potently elicits ADCC via binding to FcγRIIIa on natural killer cells (NK) and facilitates phagocytosis by other Fcγ harboring leukocytes e.g. monocyte/ macrophages and neutrophils. Hence, in addition to blockage of EGFR signaling, Cetuximab induces ADCC and the relevance of NK cells and innate immune system in the anti-cancer efficacy of this antibody has gained intense attention, also with respect to design of novel combination strategies [56–61]. Of note, NK cells as well as other components of the innate immune system are intact in the nude mice utilized in our study.

Intriguingly, the dominant transcriptional profile of UpCor genes in our study was the activation of immune response after combined ^131^I-Cetuximab and EBRT. In fact, most pathways that we found to be enriched for UpCor genes in A431 models were related to immune response processes and the gene list very much recapitulates immune activation signatures (i.e., type 1 interferon response) previously reported for other cancer agents e.g. epigenetic modulators such as DNA-methylation modifiers [62, 63]. A hub-node gene of the largest upregulated direct-interaction network (Figure 6C), ISG15, is an interferon (IFN)-stimulated gene and encodes for a ubiquitin-like protein. Similar to ubiquitin it is post-translationally conjugated to proteins by an enzymatic pathway of which the major E3 ligating component is Herc5 [64, 65]. ISG15 is mainly induced by type 1 IFNs via the Jak/STAT-pathway, induction of IFN regulatory factor (IRF) transcription factors and binding of IRFs to the IFN stimulated response element (ISRE) in the promoter of the ISG15 gene [64]. Interferon-signaling has been tightly linked to immunogenic cell death as observed under treatment with several chemotherapeutics as well as radiotherapy. Ionizing radiation may also lead to exposure of damage-associated molecular patterns (DAMP) [66]. Binding of these proteins to TLR3 or TLR4 induces IFN secretion. Subsequent signaling via Stat1 leads to the expression of ISGs [67]. Weichselbaum and colleagues have established a dual role of Stat1 with tumors constitutively overexpressing Stat1 being radioresistant while Stat1/IFN-signaling induced by radiotherapy leads to cytotoxicity [68]. Other genes in our direct interaction network have also been reported to be IFN-induced and, if overexpressed prior to radiotherapy, are associated with DNA-damage resistance such as MX1 [69]. Another interesting aspect is the appearance of RIG-I-signalling and DDX58 as the RIG-I receptor in the set of upregulated genes. RIG-I has been recently linked to the induction of IFN-signalling upon ionizing radiation via binding of small endogenous non-coding RNAs [70]. From another perspective, radiation induced DSB was very recently found to be localized to micronuclei after cellular progression towards mitosis leading to its detection by a novel double-stranded DNA sensor, cyclic GMP-AMP synthase (cGAS) and consecutive activation of stimulator of interferon genes (STING) signaling [71]. Indeed, cGAS-STING activation by radiation induced DSB and consecutive initiation of an immune and inflammatory signal is supported by a growing number of studies [72, 73]. The relevance of different recently described sensing events upstream of the radiation induced immune response remain to be elucidated.

Our data indicate that utilizing Cetuximab as an endoradiotherapy agent may benefit from radiation induced immune stimulation towards a triple, EGFR-targeting endoradiotherapy, EBRT and immune response evoking multimodal therapy. Further research may focus to reduce the long circulation time and high liver uptake of EGFR or antibody-based tumor targeting endoradiotherapy while preserving the ADCC or possibly other immunogenic properties to fully exploit the synergistic potential of carbon ions/EBRT induced IFN response and immune stimulation to eradicate tumors.

## MATERIALS AND METHODS

### Antibodies and reagents

Cetuximab (Erbitux^®^, Merck KGaA) was purchased via the in-house pharmacy. The benzamide-derivative (N-(2-diethylamino-ethyl)-4-(4-fluoro-benzamido)-5-iodo-2-methoxy-benzamide (MIP-1145)) was acquired from Molecular Insight Pharmaceuticals.

### Cell culture and mouse tumor models

Animal studies were done according to the rules for care and use of experimental animals and approved by the local and governmental Animal Care Committee instituted by the German government (Regierungspraesidium, Karlsruhe). A431 cells were purchased from ATCC (Manassas, VA, U.S.A.) and B16F10 luc2+ cells (in the following termed “B16F10”) were purchased from Caliper LS. This cell line is stably transfected with firefly luciferase gene (luc2) and thus allows for bioluminescence imaging. Both cell lines were kept in RPMI-1640 (Biochrom AG) containing 10% fetal calf serum and 1% penicilline. Cells in exponential growth phase were harvested and for the xenograft model 5 mio. A431-cells in 100 microliter PBS were injected subcutaneously in the right hind limb of 5-6 weeks old NCr nu/nu mice (Taconic). For the syngeneic model 0.25 mio. B16F10-cells in 100μl PBS were injected subcutaneously in the right hind limb of C57Bl/6 mice (Charles River Laboratories). Length and width of subcutaneous tumors were measured using a manual caliper and tumor volumes calculated using the formula (width^2^ × length) / 2.

### External beam radiotherapy

External beam irradiation was performed as described previously [74]. In brief, photon irradiation was delivered at 320 keV using a dedicated experimental irradiator (X-RAD 320, Precision X-Ray, North Branford, Connecticut, U.S.A.). Carbon ion irradiation was carried out at the Heidelberg Ion-beam Therapy center (HIT) using a pencil-beam in raster scanning technique. The irradiation field covered a Spread-out Bragg Peak (SOBP) of 10 mm at a water equivalent depth of 120 mm (245.4–257.0 MeV/u). Tumors were placed at mid SOBP. Tumors were treated at homogenous physical carbon ion dose. Local effect model (LEM, [75]) was utilized to estimate equivalent dose for photon irradiation.

### Radiolabelling, biodistribution and endoradiotherapy

Radiolabelling of Cetuximab and benzamide MIP-1145 with ^131^I was performed as described previously [37]. B16F10 animals were injected with ^131^I-Benzamide and A431 animals were injected with ^131^I-Cetuximab on day 3 after the last fraction of EBRT for biodistribution and treatment studies. For biodistribution studies animals were sacrificed at 24h p.i. and organs were excised, weighed and their respective radioactivity measured. Organ-uptake was assessed by calculating the fractional uptake of the injected dose in each organ divided by its weight (%ID/g). In B16F10 biodistribution assay was conducted in n = 11 untreated animals and n = 3-4 animals who had undergone photon- or carbon-EBRT. In A431 biodistribution assay was performed in n = 3-6 animals per group.

Endoradiotherapy was administered intravenously via the tail vein around the third day after the last fraction of external beam radiotherapy. A431-bearing animals were exposed to 7.2 MBq ± 0.2 ^131^I-Cetuximab each. B16-bearing mice were injected with 13.31 ± 1.18 MBq ^131^I-Benzamide.

For *in vivo* treatment studies in A431 n = 7-10 animals in therapy groups and n = 17 animals in control group allowing take out of size-matched tumors for histology. In B16F10 n = 6-10 animals in therapy groups and n = 11 in control group.

### Gamma camera and bioluminescence imaging

An A431-bearing mouse was injected with ^125^I-Cetuximab anaesthetized by inhalation of 1.5% Isoflurane and oxygen and imaged at several time-points post injection using a gamma camera. B16F10 luc2+-bearing mice were anaesthetized by inhalation of 1.5% Isoflurane and oxygen. 3 mg luciferine were injected intraperitoneally per mouse. The plateau phase of luciferase activity was established prior (data not shown). For the following experiments animals (n:3-6) were imaged 8 min. after injection using a Xenogen iVis 200 bioluminescence imager (PerkinElmer, Waltham, Massachussetts, U.S.A.).

### Immunohistochemistry

Animals were sacrificed and tumor tissue harvested one week after the last fraction of EBRT or 5 days after endoradiotherapy in both tumor models. Snap frozen tumor tissue was processed as described previously [74]. For both, CD31- and Ki-67-stainings of representative areas of each tumor-section were chosen excluding necrotic areas and imaged using a conventional fluorescence microscope (Zeiss CellObserver, Zeiss, Germany) at 200x magnification. Rat anti-CD31 (Dianova) for microvessel-staining and rabbit anti-Ki67 (Abcam) for labelling Ki-67 were used as primary antibodies. Donkey anti-rabbit Alexa Fluor 488 and goat anti-rat Alexa Fluor 555 (Invitrogen) served as the corresponding secondary antibodies. DAPI (Millipore) was applied for nuclear counter-staining.

Microvessel-density was evaluated by counting the number of CD31 positive vessels per high-power field automatically using ImageJ. At least 3 representative high-power fields per tumor-section were analysed in 3 animals per group.

For the assessment of the Ki-67 index of proliferating tumor cells at least 5 HPF per tumor-section in 3 animals per goup were imaged. The number of Ki-67-positive cells was counted automatically using ImageJ and divided by the number of DAPI-positive cells per HPF.

### Transcriptome analysis

Tumor-tissue for expression analysis was collected on the same day as tissue for immunohistochemistry. Total-RNA was isolated from snap-frozen tumor tissue using the RNeasy Kit (Qiagen N.V., Venlo, Netherlands) according to the manufacturers protocol. For genome-wide expression analysis Illumina HumanHT-12 v4 Expression BeadChips were used for A431 tumors and Illumina MouseWG-6 v2.0 Expression BeadChips for B16F10 tumors. For A431, tissue of n = 3 animals per group for all treatment groups was analyzed. For B16, tumors from n = 4 animals in the control group, n = 2 in the endoradiotherapy only group and n = 3 in all other groups were analyzed. Gene transcripts with 30% or more non-assessable reads on the microarray were excluded from the analysis. To further correct for background-noise from the microarray read-out, only genes with a mean intensity-level of 100 were included in the analysis which is in line with the level of background-noise according to the microarrays’ quality control protocol. After background-correction 26,307 transcripts remained of originally 48,107 transcripts on the chip in A431 and 25,321 of 57,140 transcripts remained for analysis in the case of B16 model. Data of all groups was normalized to the arithmetic mean values of the respective control-groups. An initial ANOVA of all therapy groups in A431 was performed and hierarchical clustering using Euclidian distance was performed. The resulting centroid profiles served as template for Pavlidis Template Matching (PTM) for selection of genes with similar expression pattern from the entire data set. The identified template for PTM demonstrated a gradual expression profile from endoradiotherapy only over EBRT only to combined treatment (see centroid profiles Figure 7A and 7B). Genes correlating (UpCor) or anti-correlating (DownCor) with this profile by a correlation coefficient of r ≥ 0.7 or 0.8 were then searched for gene-set enrichment in known biochemical pathways in the kyoto encyclopedia of genes and genomes (KEGG)-database using hyper-geometric distribution. Direct-interaction networks of significantly correlating genes were created by comparing to the NCBI-database of known direct protein-protein interactions. All microarray-analyses were performed using the in-house developed Software SUMO, version 1.61j. Microarray data are available online at ArrayExpress (http://www.ebi.ac.uk/arrayexpress), under the accession number E-MTAB-6514 (B16F10) and. E-MTAB-6515 (A431).

### Statistical analysis

Testing for statistical significance was performed utilizing Student's *T*-test where appropriate in Microsoft^®^ Excel 2010. Survival analysis and log-rank test were done using SUMO. Values are presented as mean ± SEM if not otherwise indicated. Results were considered statistically significant if p-value was < 0.05 and highly significant if p-value was < 0.01.

## SUPPLEMENTARY MATERIALS FIGURES


