# Supplementary material for: Combined external beam radiotherapy with carbon ions and tumor targeting endoradiotherapy
Source: Oncotarget. 2018 Jul 6;9(52):29985–30004. doi: 10.18632/oncotarget.25695 (PMC6057461; doi:10.18632/oncotarget.25695)
Supplement: Supplementary file 1 [file oncotarget-09-29985-s001.pdf]

Combined external beam radiotherapy with carbon ions and tumor targeting endoradiotherapy

SUPPLEMENTARY MATERIALS

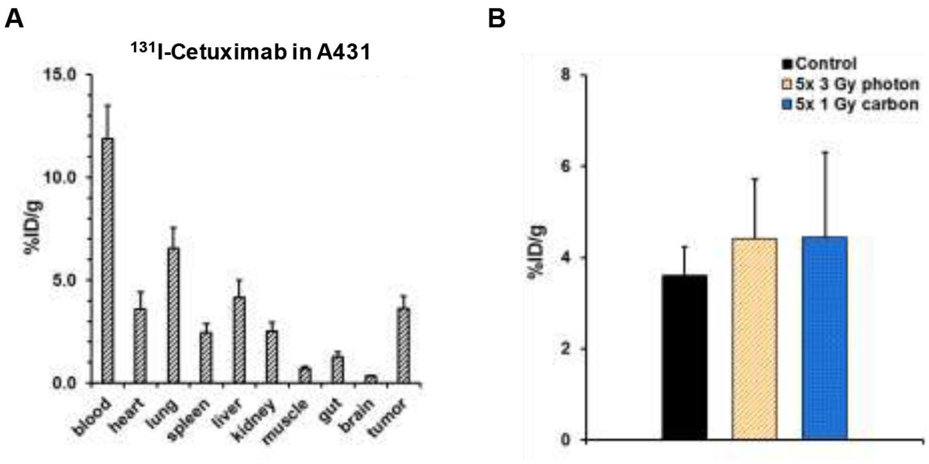

Supplementary Figure 1: *In vivo* biodistribution of ^{131}I-Cetuximab in A431-bearing nude mice. Tumor-bearing animals were injected with ^{131}I-labeled Cetuximab and organ distribution was assessed 24h thereafter **(A)** To analyze the effect of prior irradiation on tracer uptake animals underwent EBRT first and tracers were injected on the third day after the last fraction **(B)** EBRT-doses were 5x 3 Gy photon or 1 Gy carbon daily. Again, organ distribution was measured 24h after tracer injection. Data points indicate mean ± SEM *: p-value < 0.05, **: p-value < 0.01.

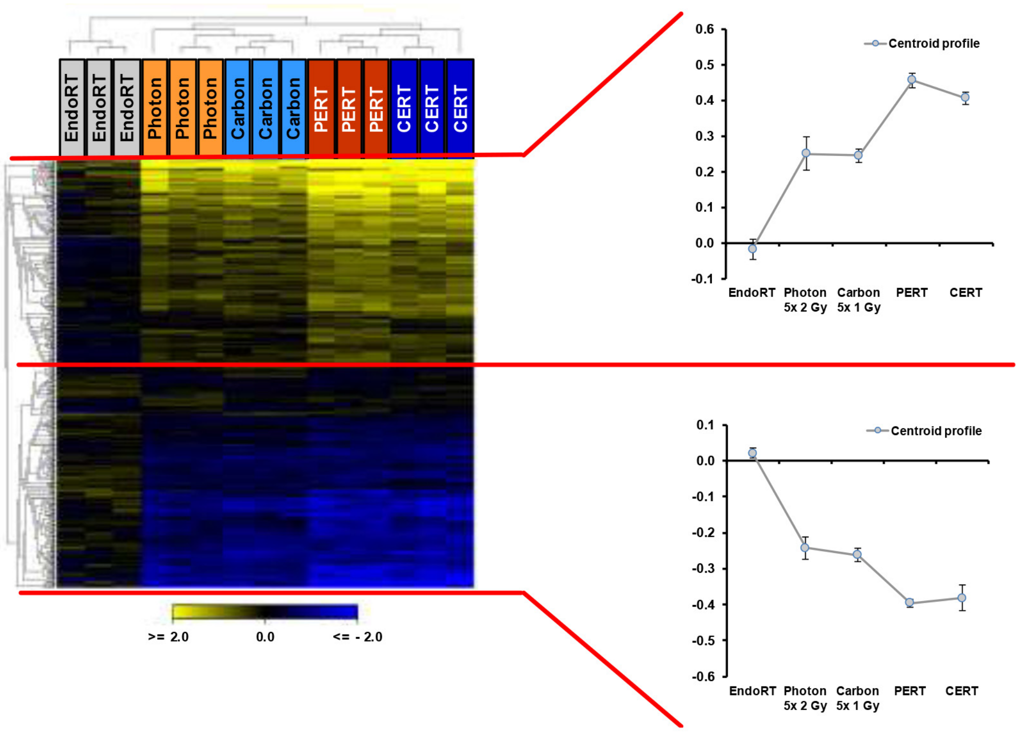

Supplementary Figure 2: ANOVA of genome-wide expression data in A431 reveals gradual gene regulation with escalation of treatment. Genome-wide expression data of A431 tumors after therapy was normalized to the control group and ANOVA performed with 5 groups of 3 samples each: EndoRT, photon-EBRT, carbon-EBRT, PERT and CERT. The ~500 most significantly regulated genes were selected ($p = 0.002$) and a Euclidian cluster analysis performed. The resulting grouping reflected the escalation of treatment from EndoRT over EBRT to combined modality treatment with correspondingly gradually increasing or decreasing centroid profiles (right). This gradual expression pattern was therefore used as template for further analyses.

A

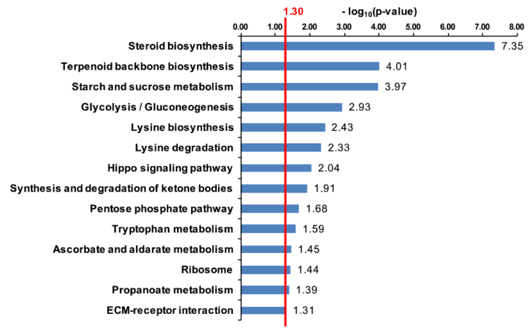

B

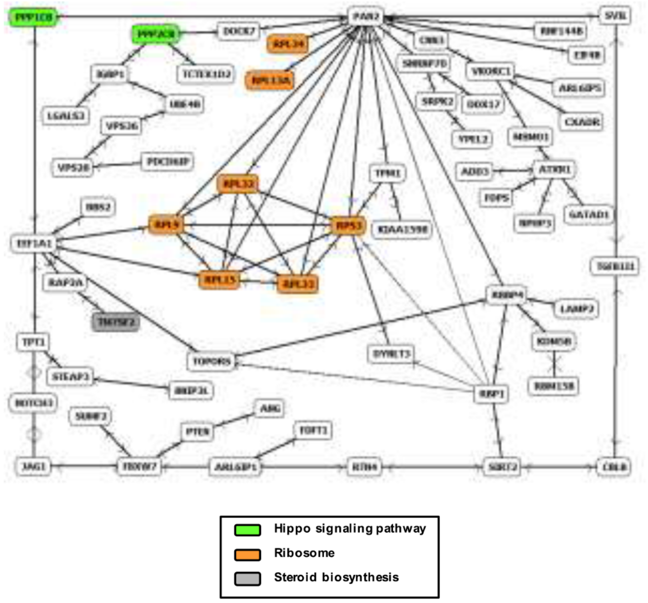

#	Node	No. of interactions
1	PAN2	17
2	RPS3	8
3	EEF1A1	7
4	RBP1	6
5	RPL15	6
6	RPL9	6
7	ATXN1	5
8	RBBP4	5
9	RPL23	5
10	RPL32	5
11	FBXW7	4
12	VKORC1	4
13	ARL6IP1	3
14	IGBP1	3
15	PPP2CB	3
16	SIRT2	3
17	SNRNP70	3
18	TOPORS	3
19	TPM1	3
20	TPT1	3
21	CBLB	2
22	CNN3	2
23	DOCK7	2
24	DYNLT3	2
25	JAG1	2
26	KDM5B	2
27	MSMO1	2
28	NOTCH3	2
29	PPP1CB	2
30	PTEN	2
31	RAP2A	2
32	RTN4	2
33	SRPK2	2
34	STEAP3	2
35	SVIL	2
36	TGFB1I1	2
37	UBE4B	2
38	VPS28	2
39	VPS36	2

Supplementary Figure 3: Anti-correlating genes in PTM-analysis of A431-microarray data. As described for the positively correlating genes microarray data of A431 tumors was searched for genes that showed a negative correlation with the predefined gradual expression profile. In this case a correlation coefficient of $r \leq -0.7$ ($p < 0.01$) was chosen as a cut-off. **(A)** The set of significantly correlating genes was compared to the KEGG-database. All significantly enriched KEGG-pathways are presented here. The red-line indicates the cut-off for statistical significance to the level of $p < 0.05$. **(B)** Networks of known direct interactions among the respective gene products were also constructed. The largest direct interaction-network is presented here. Color-coding of gene-names indicates participation in one of the significantly enriched KEGG-pathways in (A).

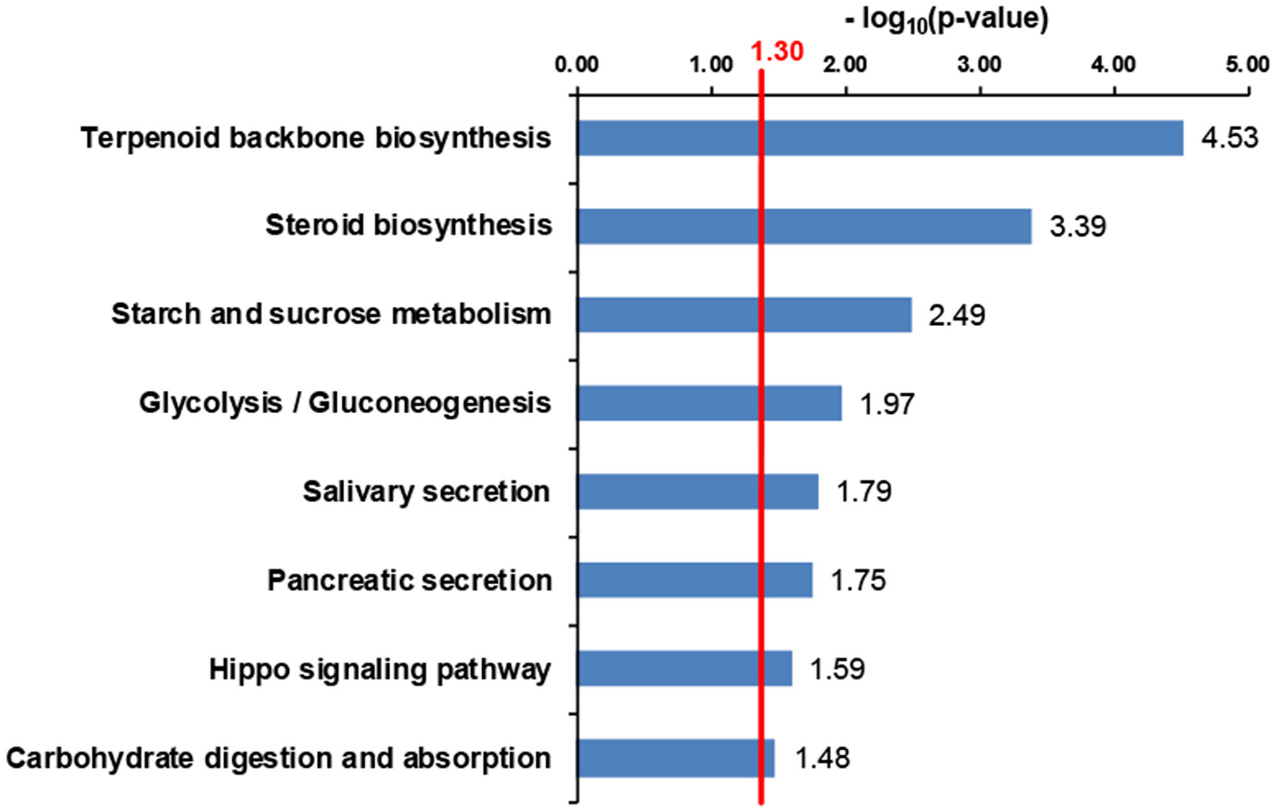

Supplementary Figure 4: Anti-correlating genes in PTM-analysis of A431-microarray data. As described for the positively correlating genes microarray data of A431 tumors was searched for genes that showed a negative correlation with the predefined gradual expression profile. In this case a correlation coefficient of $r \leq -0.8$ ($p < 0.001$) was chosen as a cut-off. The set of significantly correlating genes was compared to the KEGG-database. All significantly enriched KEGG-pathways are presented here. The red-line indicates the cut-off for statistical significance to the level of $p < 0.05$.

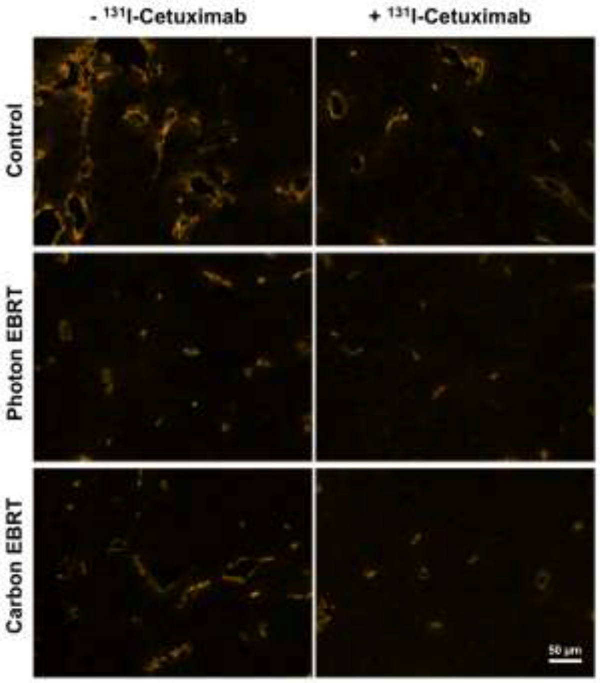

Supplementary Figure 5: Reduced microvascular density after combined EBRT and 131I-Cetuximab endoradiotherapy (CD31 only). Immunohistochemistry of tumor sections one week after EBRT or 5 days after endoradiotherapy. Microvessel density was analyzed by immunofluorescent staining for endothelial CD31 (red). Identical sections as in Figure 4 A are shown, in this case without nuclear co-staining to improve visibility of reduction in microvascular density under combined treatment with EBRT and EndoRT. Brightness of the figure as a whole has been increased to further improve visibility.
